# Neuron-specific enolase levels immediately following cardiovascular surgery is modulated by hemolysis due to cardiopulmonary bypass, making it unsuitable as a brain damage biomarker

**DOI:** 10.1007/s10047-023-01398-9

**Published:** 2023-04-29

**Authors:** Nobuya Motoyoshi, Masahiro Tsutsui, Kouji Soman, Tomonori Shirasaka, Takayuki Narita, Shingo Kunioka, Katsuyuki Naya, Daisuke Yamazaki, Masahiko Narita, Hiroyuki Kamiya

**Affiliations:** 1 Department of Clinical Engineering, Asahikawa Medical University Hospital, Asahikawa, 078-8510 Japan; 2 Department of Cardiac Surgery, Asahikawa Medical University, Midorigaokahigashi2, Asahikawa, Hokkaido 078-8510 Japan

**Keywords:** NSE, Free hemoglobin, Hemolysis, Brain disorder

## Abstract

Neuron-specific enolase (NSE) is one of the biomarkers used as an indicator of brain disorder, but since it is also found in blood cell components, there is a concern that a spurious increase in NSE may occur after cardiovascular surgery, where cardiopulmonary bypass (CPB) causes hemolysis. In the present study, we investigated the relationship between the degree of hemolysis and NSE after cardiovascular surgery and the usefulness of immediate postoperative NSE values in the diagnosis of brain disorder. A retrospective study of 198 patients who underwent surgery with CPB in the period from May 2019 to May 2021 was conducted. Postoperative NSE levels and Free hemoglobin (F-Hb) levels were compared in both groups. In addition, to verify the relationship between hemolysis and NSE, we examined the correlation between F-Hb levels and NSE levels. We also examined whether different surgical procedures could produce an association between hemolysis and NSE. Among 198 patients, 20 had postoperative stroke (Group S) and 178 had no postoperative stroke (Group U). There was no significant difference in postoperative NSE levels and F-Hb levels between Group S and Group U (*p* = 0.264, *p* = 0.064 respectively). F-Hb and NSE were weakly correlated (*r* = 0.29. *p* < 0.01). In conclusion, NSE level immediately after cardiac surgery with CPB is modified by hemolysis rather than brain injury, therefore it would be unreliable as a biomarker of brain disorder.

## Introduction

With regard to brain disorders following cardiovascular surgery, stroke and higher brain dysfunction are reported to occur at a rate of 0.8–5.2% [1] and 28–79% [2], respectively. These disorders are grave postsurgical complications that prolong intensive care unit (ICU) and hospital stay durations and increase mortality. Although it may be possible to reduce the degree of brain dysfunction through early diagnosis and the administration of pharmacotherapy, cryotherapy, or even a regenerative medicine protocol, such as stem cell therapy, in the future [3], an early diagnostic method is yet to be established.

A wide range of biomarkers are currently being investigated as indicators for head trauma, postresuscitation encephalopathy, and other brain disorders following cardiovascular surgery involving cardiopulmonary bypass (CPB). The most frequently used biomarker among them is neuron-specific enolase (NSE). In our institute, NSE has been measured routinely in all patients after cardiac surgery or patients suffered from cardiac arrest for early detection of neurological complications and its severity. Previously, we reported usefulness of serum NSE level 24 h after surgery as a predictor of neurologic outcome after aortic surgery [4]. However, as its level is normally evaluated at 24 h after an event, there is a lack of consensus regarding its appropriateness for extremely early diagnosis. NSE is known to be present in red blood cells (RBCs), platelets, and lymphocytes [5, 6]. In cardiovascular surgery, increased NSE levels as a biomarker for brain damage may be misleading as hemolysis due to transfusion or CPB is often observed. In the present study, we aimed to investigate the correlation between the NSE levels and degree of hemolysis during cardiovascular surgery and evaluate the usefulness of postoperative NSE levels for diagnosing brain damage immediately after surgery.

## Methods

### Cases

The present retrospective study was conducted with the approval of the Ethics Committee of Asahikawa Medical University Hospital (Approval number: 21048). Overall, 198 patients who underwent cardiovascular surgery with CPB between May 2019 and May 2021 at the Asahikawa Medical University hospital were included in the study. On the other hand, a study about the influence of haptoglobin administration protocol guided with free hemoglobin level on postoperative acute kidney injury in patients after cardiac surgery conducted by the department of anesthesiology in our hospital was done between April 2017 and November 2021 (Approval number: 19045). Because all patients included in the present study were also included in another study conducted by the anesthesiology, anesthesiologic and hemolysis management was determined by anesthesiologists. Moreover, patients on dialysis who had elevated NSE levels [7, 8] were excluded. Among the patients, 50 underwent aortic surgery with selective antegrade cerebral perfusion (SACP) and circulatory arrest, 15 underwent minimally invasive cardiac surgery (MICS) via right mini thoracotomy, 67 were valve cases, 40 were coronary artery bypass graft (CABG) cases, 16 were CABG + valve cases, and 10 were other cases.

The patients’ characteristics as well as perioperative and postoperative data were obtained from their medical records.

### Cardiopulmonary bypass techniques

Before cannulation, the patients were administered with 300 IU/kg heparin, and CPB was initiated when activated clotting time was observed to be ≥ 480 s. The machines used in CPB were an S5 heart–lung machine (LivaNova, London, UK) and centrifugal pump (Revolution, LivaNova). For all the patients, a membrane oxygenator (CAPIOX FX-15 or FX-25, terumo; Affinity Fusion, Medtronic) was used with the open-circuit method. The priming volume was 800–1300 ml (VOLUVEN 6% solution for infusion, FRESENIUS KABI JAPAN; BICANATE Injection, Otsuka; mannitol injection 0.4 g/kg, Yoshindo). The patients were then administered with an additional 100 IU/kg of heparin. Blood was collected from all the patients using vacuum-assisted venous drainage with − 10 to − 30 mmHg of negative pressure. For the patients undergoing MICS, a negative pressure of − 60 mmHg was permitted. During the CPB, a mild hypothermia at 34 °C was induced. Pump flow was managed at 2.2–2.6 ml/min/m^2^, mixed venous oxygen saturation target was ≥ 75%, and perfusion pressure was maintained at 40–80 mmHg. The alpha-stat strategy was used to manage pH. Hematocrit levels were maintained at ≥ 21% during the CPB, and RBC transfusions were administered when it dipped below this level. A moderate hypothermia at 24–26 °C was induced for circulatory arrest. SACP was used for cerebroprotection with a perfusion rate of 10–13 ml/kg/min. The perfusion pressure was maintained at 30–50 mmHg. The residual blood was returned to the body whenever possible, and the remaining blood was returned using an autotranfusion system (XTRA, LivaNova).

### Measurement of free hemoglobin and administration of haptoglobin preparations

Free hemoglobin (F-Hb) was measured using the Hemocue® Plasma/Low Hemoglobin system (HemoCue AB, Angelholm, Sweden). From each patient, 2 ml of blood was collected and centrifuged for 10 min at 3,652 rpm in a Kubota 2420 tabletop centrifuge. The resulting plasma was drawn into the specialized Hemocue® dropper and F-Hb was measured. F-Hb was measured every 60 min of the CPB and after the CPB ended.

Regarding dosage standards, 2000 and 4000 units of haptoglobin preparation (Hp) were administered if the F-Hb levels were ≥ 30 and ≥ 50 mg/dl, respectively. When applicable, these were administered every 60 min of the CPB. When the F-Hb level was measured again after the CPB was removed, Hp was administered according to the same criteria. There was no upper limit placed for Hp administration according to the protocol by anesthesiologists.

### Examination method

To investigate the usefulness of NSE levels immediately following surgery in diagnosing brain damage, the postoperative NSE levels of the group without postoperative stroke was compared with those of the group with postoperative stroke. Furthermore, the F-Hb levels were compared between the two groups. The F-Hb levels used were those measured after the CPB ended. To investigate the relationship between hemolysis and NSE, the correlations between the NSE and F-Hb levels and between the NSE and lactate dehydrogenase (LDH) levels were evaluated. The F-Hb levels used were those measured after the CPB ended, and the LDH and NSE levels used were those measured when the patients returned to the ICU. Moreover, we investigated whether there was a correlation between the CPB time and NSE levels. To determine whether there was an association between NSE and hemolysis from the different surgical procedures, we broadly categorized the cases according to the procedures received by the patients and investigated the correlations. The cases were classified as follows: 50 aortic surgical cases, 15 MICS cases, and 133 other cases (67 valve cases, 40 CABG cases, 16 CABG + valve cases, and 10 other cases as follows 3 ventricular septal defect closure cases, 2 ventricular perforation closure cases, 2 left ventricular repair cases, 2 cardiac tumor resection cases and 1 pulmonary vein stenosis release case). The rate of F-Hb level increase was calculated with a linear regression comparing F-Hb (y, mg/dl) and CPB time (x, min) (the F-Hb level was measured every 60 min at 467 points).

### Diagnosis of postoperative stroke

Patients presented neurological abnormality, e.g., severe delirium, convulsion, paralysis or coma were evaluated by computed tomography or magnetic resonance imaging. The diagnosis of stroke was done by neurologists by reference to such neurological imaging.

### Statistical techniques

Patients’ background characteristics, intraoperative parameters, postoperative test values, etc. were expressed as mean ± standard deviation (SD). The two groups were compared using Mann–Whitney U test. Pearson correlation was used to determine the relationship between hemolysis and NSE, with a r of > 0.2– ≤ 0.4, 0.4– ≤ 0.7, and > 0.7 indicating weak, moderate, and strong correlations, respectively. The statistical software used was Pharmaco Basic Ver 16 (Scientist Press Co., Ltd, Tokyo, Japan).

## Results

### Comparison of the NSE levels between patients with and without stroke

**Fig. 1:**
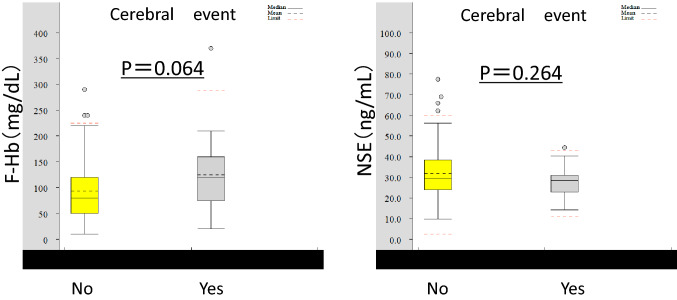
Comparison of the NSE and F-Hb levels between the groups with and without stroke. *F-Hb* free hemoglobin, *NSE* neuron-specific enolase

Among the participants, 178 (Group U) did not experience postoperative neurological damage and 20 (Group S) were diagnosed with stroke by computed tomography or magnetic resonance imaging. Of the 20 patients with stroke, 13 had disabling stroke, 4 had minor stroke that improved with continued rehabilitation, and 3 exhibited no symptoms or quickly recovered without permanent damage. The patients’ background characteristics, surgical procedures, and CPB times in relation to the presence or absence of brain damage are shown in Table 1. A statistically significant difference in the patients’ demographic data, including hypertention, aortic surgery, and CPB time, was observed between the groups with and without stroke. The mean NSE levels between the groups without neurological dysfunction (Group U, *n* = 178) and with stroke (Group S) were not significantly different (31.8 ± 11.2 ng/ml [Group U] vs 28.5 ± 7.6 ng/ml [Group S]); *p* = 0.264). The F-Hb levels were 93.8 ± 54.8 and 125 ± 79.2 mg/dl in the Group U and S, respectively, showing no significant difference (*p* = 0.064) (Fig. 1).

**Table 1:** Comparison of patient background, surgical procedure, and surgical parameters between the patients with and without postoperative brain damage

	Uncomplicated (*n* = 178)	Stroke (*n* = 20)	*P* value
Age (years), mean ± SD	70.5 ± 12.8	70.5 ± 11.8	*P* = 0.95
Male sex, *n* (%)	117 (66)	11 (55)	*P* = 0.34
BMI (kg/m^2^), mean ± SD	23.6 ± 3.7	24.7 ± 3.9	*P* = 0.21
Hypertension, *n* (%)	119 (67)	18 (90)	*P* = 0.03
Diabetes mellitus, *n* (%)	50 (28)	5 (25)	*P* = 0.77
Hyperlipidemia, *n* (%)	92 (52)	9 (45)	*P* = 0.57
s-Creatinine (mg/dl), mean ± SD	1.02 ± 0.47	0.96 ± 0.36	*P* = 0.86
Aortic surgery, *n* (%)	38 (21)	12 (60)	*P* = 0.00016
MICS, *n* (%)	15 (9)	0 (0)	*P* = 0.16
Others, *n* (%)	125 (70)	8 (40)	*P* = 0.006
CPB time (min), mean ± SD	157 ± 64	208 ± 101	*P* = 0.01
Aortic clamp time (min), mean ± SD	109 ± 44	117 ± 50	*P* = 0.52

*BMI* body mass index, *CPB* cardiopulmonary bypass, *MICS* minimally invasive cardiac surgery

### Correlation between NSE and degree of hemolysis

**Fig. 2:**
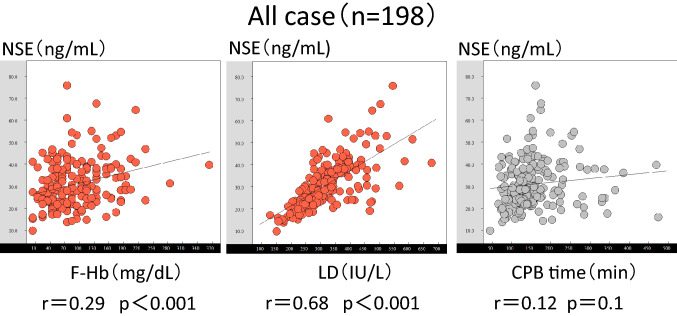
Graph of the correlations between the F-Hb and NSE levels, LDH and NSE levels, and CPB time and NSE levels in the patients. *F-Hb* free hemoglobin, *NSE* neuron-specific enolase, *LDH* lactose dehydrogenase, *CPB* cardiopulmonary bypass

The relationships between the F-Hb and NSE levels, the LDH and NSE levels, and CPB time and NSE levels are shown in Fig. 2. A weak correlation was observed between the F-Hb and NSE levels (*r* = 0.29, *p* < 0.001). A moderate correlation was observed between the LDH and NSE levels (*r* = 0.68, *p* < 0.001). No correlation was observed between the CPB time and NSE levels (*r* = 0.12, *p* = 0.1).

### Amount of used haptoglobin

Total amount of administrated haptoglobin was 9520 ± 3649 units in patients underwent aortic surgery (*n* = 50), 1866 ± 2875 units in patients underwent MICS (*n* = 15), and 6812 ± 4207 units in patients underwent other surgeries (*n* = 133), and there was significant difference between each group (*p* = 0.0001). In group S and group U, the total amount of administrated haptoglobin was 9600 ± 5255 and 6464 ± 4151 units, respectively (*p* = 0.002).

### Correlations between the NSE levels and hemolysis for different surgical procedures

**Fig. 3:**
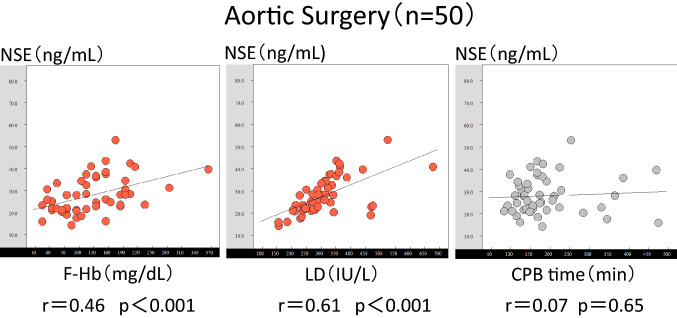
Graph of the correlations between the F-Hb and NSE levels, LDH and NSE levels, and CPB time and NSE levels in the aortic surgery cases. *F-Hb* free hemoglobin, *NSE* neuron-specific enolase, *LDH* lactose dehydrogenase, *CPB* cardiopulmonary bypass

**Fig. 4:**
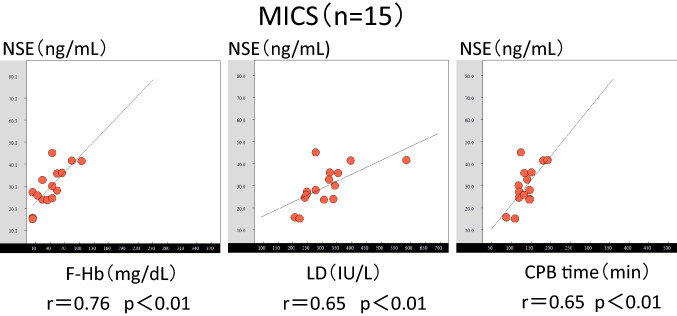
Graph of the correlations between the F-Hb and NSE levels, LDH and NSE levels, and CPB time and NSE levels in the MICS cases. *F-Hb* free hemoglobin, *NSE* neuron-specific enolase, *LDH* lactose dehydrogenase, *CPB* cardiopulmonary bypass, *MICS* minimally invasive cardiac surgery

**Fig. 5:**
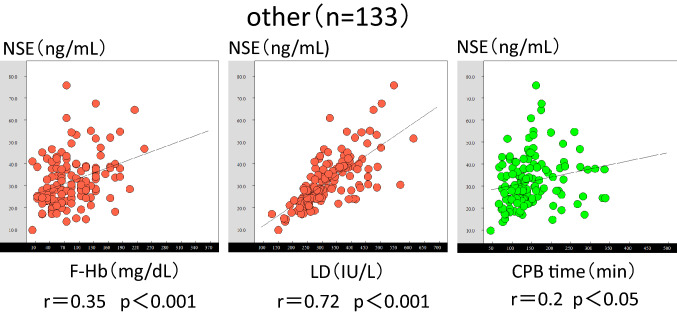
Graph of the correlations between the F-Hb and NSE levels, LDH and NSE levels, and CPB time and NSE levels in the patients who underwent other procedures. *F-Hb* free hemoglobin, *NSE* neuron-specific enolase, *LDH* lactose dehydrogenase, *CPB* cardiopulmonary bypass

The relationships for the aortic surgery group are shown in Fig. 3. A moderate correlation was obtained with the F-Hb and NSE levels (*r* = 0.46, *p* < 0.001). Moreover, a moderate correlation was obtained with the LDH and NSE levels (*r* = 0.61, *p* < 0.001). No correlation was observed between the CPB time and NSE levels (*r* = 0.07, *p* = 0.65). The relationships for the MICS group are shown in Fig. 4. There was a strong correlation between the F-Hb and NSE levels (*r* = 0.76, *p* < 0.01) and a moderate correlation between the LDH and NSE levels (*r* = 0.65, *p* = 0.01). A moderate correlation was observed between the CPB time and NSE levels (*r* = 0.65, *p* < 0.01). The relationships for the other cases are shown in Fig. 5. A weak positive correlation was observed between the F-Hb and NSE levels (*r* = 0.35, *p* < 0.001). A strong positive correlation was observed between the LDH and NSE levels (*r* = 0.72, *p* = 0.001) A weak positive correlation was observed between the CPB time and NSE levels (*r* = 0.2, *p* < 0.05). The average rate of F-Hb level increase during the CPB was 0.37 mg/dl/min (*p* < 0.001) according to the calculated regression line, *y* = 0.37x + 40.1.

## Discussion

In the present study, the hemolysis indicator F-Hb displayed a weak correlation with the NSE levels. Conversely, the LDH levels displayed a strong correlation with those of NSE. The NSE levels tended to increase as hemolysis proceeded. No correlation was observed between the CPB time and NSE levels. When the patients were analyzed in terms of the surgical procedures they underwent, a strong correlation between the F-Hb and NSE levels was observed in the MICS group. Differences in the correlation between hemolysis and the NSE levels were observed among the different surgical procedures. There was no difference in the NSE levels between the groups with and without neurological dysfunction; thus, we considered it to be difficult to objectively assess brain damage immediately following surgery using NSE levels as a biomarker (Table 2).

**Table 2:** NSE levels and stroke in different surgical techniques

	Without stroke	With stroke	*P* value
Aortic surgery (*n* = 50)	28.6 ± 8.9 (*n* = 38)	27.9 ± 8.3 (*n* = 12)	0.95
Others (*n* = 133)	32.9 ± 11.9 (*n* = 125)	29.3 ± 6.8 (*n* = 8)	0.49
MICS (*n* = 15)	30.3 ± 9.3 (*n* = 15)	NA	NA

*MICS* minimally invasive cardiac surgery, *NA* not available

### NSE as a biomarker for brain damage following cardiovascular surgery

Regarding the correlation between the NSE levels and neurological complications following cardiac surgery, while NSE was reported to be a useful biomarker for brain damage [9, 10], Ishida et al. [11] stated that, in mediastinal surgery, since a significant amount of NSE is present in circulation, this mixes with the cardiotomy suction blood, which makes the use of NSE levels unreliable for measuring brain complications. Considering that the biological half-life of NSE is 48 h, it is possible that its level at ≥ 2 days after surgery may reflect brain damage conditions. We considered this as one of the reasons as to why no significant difference was observed in the NSE levels immediately after surgery between the groups with and without brain damage.

### Hemolysis during cardiovascular surgery

Hemolysis is frequently observed during cardiovascular surgeries. In most cases, it is caused by CPB, RBC transfusion, or blood salvage, and triggers an increase in the F-Hb level [12]. Cheung et al. reported that the F-Hb levels increased with increasing CPB time at a rate of 0.27 mg/dl/min [13], which was similar to our results (0.37 mg/dl/min).

### NSE levels and degree of hemolysis

When hemolysis is triggered in vitro, the increase in the NSE levels shows a good correlation (*r*^2^ = 0.953) with the degree of hemolysis, and the NSE levels increase by 0.29 ng/ml for every 1-mg/dl increase in F-Hb concentration [14]. Our results indicated a 0.06 ng/ml increase for every 1-mg/dl increase in F-Hb concentration. We considered that in vivo the NSE levels increase more gradually with respect to the F-Hb levels as compared with that observed in in vitro studies due to the influence of metabolic pathways, such as that of haptoglobin, and Hp administration. Although Johnsson et al. reported a correlation coefficient of 0.55 for F-Hb and NSE levels [15], in the present study, the correlation was weaker (*r* = 0.29). We consider that this difference can be attributed to factors such as a decrease in the F-Hb levels because of administering repeated doses of Hp during the CPB without an upper limit. Concurrently, a correlation between the NSE levels and degree of hemolysis was observed. Hence, we concluded that the NSE levels are influenced by the degree of hemolysis.

### Relationship between Haptoglobin and F-Hb

When cardiovascular surgery with CPB begins, the haptoglobin and F-Hb levels starts to decrease and increase, respectively, and they reach their respective minimum and maximum values after the CPB ends [16, 17] There have been a few reports on the kinetics of F-Hb after haptoglobin administration. Tanaka et al. administered haptoglobin when the F-Hb levels exceeded 30 mg/dl during the CPB; they found that the F-Hb concentration disappeared in nearly all the cases following haptoglobin administration [18]. However, they did not mention the measurements of the F-Hb levels or hemolysis effects from the subsequent CPB. In the present study, the F-Hb levels were measured hourly during the CPB and once after its removal. If the F-Hb levels were ≥ 30 and ≥ 50 mg/dl, 2000 and 4000 units of Hp were administered, respectively. However, in nearly all the cases, the F-Hb levels continued to increase even after the CPB ended despite the administration of haptoglobin. However, it is possible that repeatedly administering haptoglobin from mid-CPB onwards with no upper limit reduced the F-Hb levels compared with what it might have been without Hp administration. We considered this to be the reason for the weak correlation between the NSE and F-Hb levels and a strong correlation between the NSE and LDH levels.

### Difference in the degree of hemolysis among the different surgical procedures

Windsant et al. reported that the F-Hb levels were higher in the CABG + valve surgery compared with that in CABG alone [19]. They suggested that the F-Hb levels increased in the CABG + valve surgery due to the enhanced complexity of this surgical procedure, which involves longer CPB time, higher transfusion volumes, and frequent suctioning, thereby damaging the RBCs. Paparella et al. reported that the F-Hb levels are significantly lower in the patients who underwent MICS than in those who underwent medial incision [20].

Similarly, we found that the F-Hb levels were the highest in the patients who underwent aortic surgery, followed by “Others,” and the lowest levels were found in those who underwent MICS. This phenomenon was also seen in the amount of administrated haptoglobin reflecting the degree of hemolysis in different procedures; aortic surgery > other procedures > MICS. Among the types of surgical procedures, the correlation between the F-Hb and NSE levels was the strongest in the patients who underwent MICS. We considered this to be because in MICS, which involves minimal bleeding and requires the lowest transfusion volumes [21], a small amount of high-NSE blood collected from the mediastinal area [11], there are a few factors causing hemolysis other than the mechanical hemolysis caused by the CPB. We also considered the prolongation of the CPB in MICS to have caused rise in the NSE levels.

### Possibility of NSE as a brain damage biomarker when hemolysis is accounted for

During the CPB, hemorrhage and transfusion cause hemolysis. As suction in the mediastinal area may affect the NSE concentration, NSE levels are an unreliable biomarker of brain damage immediately following cardiac surgery. Hence, further investigation regarding NSE concentration following cardiac surgery is warranted. On the other hand, because hemolysis modulates NSE levels as demonstrated in the present study, one should be aware that NSE value may be unreliable neurological marker in patients treated with mechanical circulatory support. Based on the present study, we have stopped routine measuring NSE immediately after cardiac surgery.

### Study limitations

Some limitations need to be considered when interpreting the present results. First, the study was retrospective and could not exclude the biases inherently associated with this study type. Second, the sample size in the present study was very small, especially regarding to MICS surgery. Nevertheless, since there have been no prior reports on unreliability of NSE level as a biomarker of neurological complications immediately after cardiac surgery and its mechanisms, the present study may carry worth and meaning for further investigations.

## Conclusion

NSE level immediately after cardiac surgery with CPB is modified by hemolysis rather than brain injury, therefore it would be unreliable as a biomarker of brain disorder.
